# Rapid Enzyme-Linked Immunosorbent Assay for the Detection of Hantavirus-Specific Antibodies in Divergent Small Mammals

**DOI:** 10.3390/v6052028

**Published:** 2014-05-06

**Authors:** Karla Cautivo, Tony Schountz, Mariana Acuña-Retamar, Marcela Ferrés, Fernando Torres-Pérez

**Affiliations:** 1 Instituto de Biología, Pontificia Universidad Católica de Valparaíso, Valparaíso 2373223, Chile; E-Mail: kcautivor@gmail.com; 2 Department of Microbiology, Immunology and Pathology, Colorado State University, Fort Collins, CO 80523, USA; E-Mail: tony.schountz@colostate.edu; 3 Facultad de Cs Veterinarias y Pecuarias, Universidad de Chile, Santiago 8820808, Chile; E-Mail: mariana.acuna@gmail.com; 4 Laboratorio de Infectología y Virología Molecular, Escuela de Medicina, Pontificia Universidad Católica de Chile, Santiago 8330024, Chile; E-Mail: mferres@med.puc.cl

**Keywords:** Andes virus, Chile, ELISA, strip immunoblot assay, hantavirus

## Abstract

We assessed the utility of an enzyme-linked immunosorbent assay (ELISA) for the detection of hantavirus-specific antibodies from sera of *Oligoryzomys longicaudatus*, the principal reservoir of Andes virus (ANDV), using an antigen previously developed for detection of antibodies to Sin Nombre virus (SNV) in sera from *Peromyscus maniculatus*. The assay uses a protein A/G horseradish peroxidase conjugate and can be performed in as little as 1.5 hours. Serum samples from *Oligoryzomys longicaudatus* collected in central-south Chile were used and the assay identified several that were antibody positive. This assay can be used for the rapid detection of antibodies to divergent hantaviruses from geographically and phylogenetically distant rodent species.

## 1. Introduction

Hantaviruses are trisegmented, negative-stranded RNA viruses of the family *Bunyaviridae*. The small (S), medium (M) and large (L) segments encode the nucleocapsid (N), glycoproteins (Gn and Gc), and RNA-dependent RNA polymerase, respectively. Since the first report of hantavirus cardiopulmonary syndrome (HCPS) in 1993 in the Americas, an increasing number of hantavirus species have been identified as human pathogens [1]. Of these, Sin Nombre virus (SNV) and Andes virus (ANDV) are the most important etiologic agents in North and South America, respectively [2]. Pathogenic hantaviruses are hosted by rodents and transmitted to humans through incidental exposure to rodent secretions or excretions [3,4]. However, distinct hantaviruses of unknown pathogenicity have also been identified in several other rodent species, and in species of the orders Soricomorpha and Chiroptera [5]. 

ANDV is found in Chile and Argentina [6] (but see [7]), and differs from other hantaviruses because person-to-person, including nosocomial, transmission has been documented [8,9]. In Chile, cases of HCPS are concentrated in the central and southern regions (32°S–46°S; [10]) which include the eco-regions Mediterranean, Valdivian Temperate and Patagonian. Molecular analysis suggests that human infections are caused by a single genotype of ANDV that varies from North to South [11]. The principal reservoir of ANDV is the cricetid *Oligoryzomys longicaudatus* (long-tailed pigmy rice rat), which inhabits foothills and rural areas, and also near waterways from latitude 28°S to 55°S in Chile and south-central areas of Argentina [12,13,14]. Spill-over is presumed to have occurred to other rodent species [11,15,16], but the role of other, non-reservoir species in the transmission cycle of ANDV remains unknown. Therefore, it is useful to develop new tools for the detection of infected rodents under field and laboratory conditions.

Tests for identifying seropositive rodents have been developed [17,18], and implemented in Chile and Argentina [15,19,20]. Recently, an immune assay for detecting antibodies to SNV in *Peromyscus maniculatus* was developed in North America [21]. SNV is phylogenetically divergent from ANDV [11,22]. However, a conserved B cell epitope is found in the N terminus of New World hantavirus nucleocapsids and a truncated antigen (15 kD) containing this epitope is reactive with antibodies to several hantaviruses, including SNV, Calabazo virus [23], El Moro Canyon virus and Maporal virus [24]. Unlike other assays that use species-specific polyclonal antibodies (typically produced in rabbits or goats), this assay was developed to detect antibodies to New World hantaviruses from many mammalian species because it uses a protein A/G conjugate and is easily implemented in both the laboratory and the field, producing results in about 1.5 hours [21]. Using samples of wild rodents of south central Chile, we tested the hypothesis that the assay is useful for detecting antibodies to ANDV from multiple rodent species. We detected hantavirus-specific antibodies in two species, *Oligoryzomys longicaudatus* and *Abrothrix longipilis*, demonstrating the utility of this assay for identifying hantavirus-infected rodents.

## 2. Results

**Figure 1:**
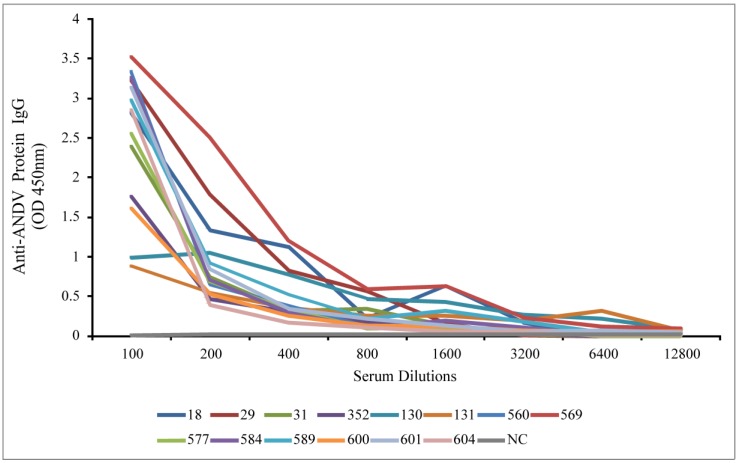
Quantitative enzyme-linked immunosorbent assay (ELISA) results. The graphics shows variations of optical density (O.D.) values measured in seropositive samples at different dilutions of sera. NC: Negative control.

A total of 282 small mammals were captured at six localities during the sampling period November 2011-July 2013. We captured 168 *Oligoryzomys longicaudatus*, 54 *Abrothrix longipilis*, 42 *Abrothrix olivaceus*, 13 *Rattus rattus*, 2 *Rattus novegicus*, 1 *Mus musculu*s, 1 *Loxodontomys micropus*, and 1 *Dromiciops gliroides* (Table 1). Fourteen serum samples were seropositive to SNV N antigen using a +4 colorimetric system, representing 5% of the total small mammals captured. Of the 14 seropositives, 13 were *O. longicaudatus* (7.73% of the total captured) and 1 *A. longipilis* (1.85% of the total captured). SIA results showed 13 rodent samples that were seropositve to antibody against ANDV. All seropositives found with ELISA except one sample (sample 560 from Lanco, Region XIV; see Figure 1 and Table 1) were seropostives using SIA.Also, two samples (from Toltén, Region IX; Table 1) were seropositive by SIA and seronegative by ELISA. Spectrophotometry analysis showed that all samples were registered seropositives in dilutions ranging from 1:100 to 1:1600 (titers were expressed as the reciprocal of the dilution that yields a positive result; see Table 2). O.D. maximum values were found in *A. longipilis* UCK569 (O.D. = 3.5156), and *O. longicaudatus* UCK577 (O.D. = 3.3285). Samples 130 and 131 showed the lowest O.D. values (1.0504 and 0.8749, respectively; Figure 1). 

**Table 1:** Sampled sites, and seropositivity of rodent species captured in the sampling period (2011–2013).

Locality	Species	Number of captures	Number of seropositives (ELISA)
Villarica, Region IX 39°25'S; 71°45'W	*O. longicaudatus* *A. olivaceus* *A. longipilis*	6 2 4 0	3 0 0
El Sauce, Chimbarongo, Region VI 70°59'W; 34°48'S	*O. longicaudatus* *A. olivaceus*	2 1	1 0
Rupanquito, Puerto Octay, Region X 40°46'S; 72°46'W	*O. longicaudatus* *A. olivaceus* *A. longipilis* *R. rattus* *R. norvrgicus* *M. musculus*	28 13 3 2 1 1	2 0 0 0 0 0
Boroa, Toltén, Region IX 39°17'S; 73°04'W	*O. longicaudatus* *A. olivaceus* *R. rattus* *R. norvrgicus*	19 1 11 1	0 0 0 0
Pichi-Juan, Pto. Varas, Region X 41°14'S; 72°43'W	*O. longicaudatus* *A. olivaceus* *A. longipilis* *L. micropus*	32 11 2 1	0 0 0 0
Miraflores, Lanco, Region XIV 39°32'S; 72°37'W	*O. longicaudatus* *A. olivaceus* *A. longipilis*	59 7 5	7 0 1
Llacolén, Contulmo, Region VII 37°54'S; 73°16'W	*O. longicaudatus* *A. olivaceus* *A. longipilis* *D. gliroides*	22 7 4 1	0 0 0 0

**Table 2:** Titers (expressed as the reciprocal of the O.D. dilution that yields a positive result) of seropositive samples to hantavirus in Chile.

Accession	Species	Sex	Age	Titer
18	*O. longicaudatus*	Female	Adult	1600
29	*O. longicaudatus*	Male	Adult	800
31	*O. longicaudatus*	Male	Adult	800
352	*O. longicaudatus*	Female	Adult	400
130	*O. longicaudatus*	Male	Adult	6400
131	*O. longicaudatus*	Male	Adult	6400
560	*O. longicaudatus*	Female	Adult	400
569	*A. longipilis*	Male	Adult	3200
577	*O. longicaudatus*	Male	Adult	400
584	*O. longicaudatus*	Male	Adult	400
589	*O. longicaudatus*	Male	Adult	800
600	*O. longicaudatus*	Male	Adult	400
601	*O. longicaudatus*	Female	Adult	800
604	*O. longicaudatus*	Male	Adult	200

## 3. Discussion

In this study, we evaluated the utility of previous methodology [21] to detect anti-hantavirus antibodies in wild rodents in Chile. Schountz et al. reported the ELISA test based on data from serum samples of deer mice (*P. maniculatus*), and suggested that the test may be useful for other small mammals harboring hantaviruses. Our results confirm the test is useful to detect anti-hantavirus antibodies in phylogenetically divergent hantaviruses such as SNV and ANDV. All samples scored seropositive by the colorimetric procedure were corroborated by spectrophotometry [21]. 

Alternative and more specific methods have been proposed to detect viral RNA and anti-hantavirus antibodies in rodents from South America [18,25,26,27]. For example, Padula *et al.* (2000) [18] developed a conventional ELISA test to detect IgM in human serum, and also stated that detection of IgG antibodies is particularly useful for serological surveys of rodents for understanding to the ecology of hantaviruses. While protein-A/G binds to human IgM, it does not bind to IgM from the laboratory house mouse (*Mus musculus*). It also moderately binds to IgA and IgE of *M. musculus*. Although we have no data on IgM (or IgA or IgE; [28]) from the long-tailed pygmy rice rat, we believe it is unlikely that this assay detects IgM. This property indicates that we are probably overlooking rodents with acute infections (a period when IgG antibody titers are expected to be lower), and that all seropositive rodents were in the chronic stage of infection. 

To determine specificity, a strip immunoblot assay (SIA) was previously developed [17], which has been used in a number of studies [11,13,15,29,30]. We used the standard SIA to determine the number of false positive or negatives. The comparison of both assays to detect rodent sample seropositives was highly congruent (13 samples resulted seropositive by SIA and 14 were seropositive by ELISA, representing a 93% specificity). These results suggest that one sample may be considered false positive using the ELISA, or false negative using the SIA. Also, two samples were seropositive by SIA and seronegative by ELISA, suggesting false negatives using ELISA. Although enzyme immunoassays are widely used to detect anti-hantavirus antibodies, immunoblot assays seem to perform better and have greater specificity than enzyme-linked immunosorbent assays [31]. Therefore, we may preliminarly consider our results as including one false positive and two false negatives using the ELISA. However, our data do not allow us to definitively discriminate between the false positive/false negative sample, and additional screening such as RNA detection by PCR should be performed (but see caveats in [32]). On the other hand, our results showed cross-reactivity of ANDV and SNV antigens in a few samples using SIA assay (see Supplementary Figure S1). The cross‑reactivity observed is likely a result of the sequence similarities of ANDV and SNV N proteins within certain identified epitopes, which may also cross-react with old world hantaviruses [33].

Despite disadvantages of enzyme-linked immunosorbent assays [31], protein A/G ELISA has the advantage that can be easily implemented for field studies with results obtained within 2 hours. New analyses evaluating precision and sensitivity in detecting seropositive small mammals should be fully explored in the future.

## 4. Experimental Section

**Figure 2:**
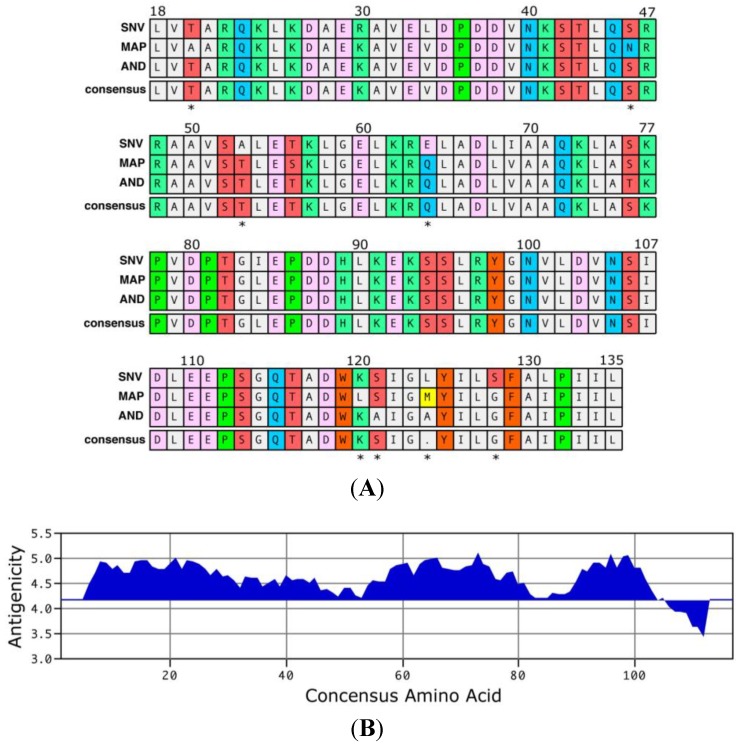
Alignment (**A**) and antigenicity (**B**) plot of the truncated nucleocapsid antigen. The 118 residue truncated Sin Nombre virus (SNV) polypeptide (15 kD) represents about 30% of the full‑length nucleocapsid and is missing the N-terminal 13 amino acids and the C-terminus. The sequence was aligned to the homologous sequences of Andes and Maporal viruses using the CLUSTALW algorithm of MacVector software. Amino acid similarities are denoted by block color. The numbers above the sequences indicate the amino acid position within the full-length nucleocapsid protein. The asterisk (*) below the consensus indicates a dissimilar residue; the overall similarity is 93% (110/118), with four of the 8 dissimilar residues found in the C-terminus region with a predicted low antigenicity index. Antigenicity prediction was performed with the protrusion index prediction algorithm of MacVector, which scores amino acids for their hydrophilic and hydrophobic characteristics and is weighted based upon those characteristics of neighboring residues. Regions of hydrophilicity (potential antibody epitopes) are positive values, while regions of hydrophobicity are negative values.

A total of 282 rodents were collected from seven localities in Chile: Chimbarongo (Region VI), Contulmo (Region VI), Boroa (Region IX), Villarrica (Region IX), Rupanquito (Region X), Puerto Varas (Region X) and Lanco (Region XIV). Rodents were captured in live traps according to standard protocols as previously described [15] and followed established safety guidelines for rodent captures and processing [34,35]. Rodent antibody against hantavirus was detected in blood samples following the method described in Schountz *et al.* (2007) [21]. Briefly, microtiter plates (96-well polyvinylchloride, Falcon 353912/353913, BD Biosciences) were coated with 1 μg/mL of recombinant truncated SNV nucleocapsid antigen in 100 μL of PBS and incubated for 16 hours at 4 °C. The plates were washed 3 times with PBS using a squirt bottle, blotted on a paper towel, and then added 200 μL of gelatin blocking buffer (0.25% in PBS) and incubated at room temperature for 1 hour. The plates were washed three times with PBS TWEEN-20 (0.5%) and blotted again. Serum samples were diluted 1:100 in 1 mL phosphate buffered saline (PBS) and 100 μL of each sample was added to the wells. After 1 hour of incubation, 100 μL of protein-A/G-HRP conjugate (Pierce Protein Biology Products 32490) diluted 1:5000 in PBS was added to each well, and incubated for 45 min. The plates were washed four times with PBS-TWEEN. Finally, 100 μL of TMB substrate was added allowing 10–15 min to react with HRP. The reaction was stopped by adding 100 μL of sulfuric acid 1N. Samples were scored as seropositves following the colorimetric procedure as previously described (Schountz *et al.* 2007) [21]. Optical density was measured at a wavelength of 450 nm using a spectrophotometer (Versamax^® ^Microplate Reader, Molecular Devices). Samples that were seropositive were diluted 1:100 to 1: 12800 to determine endpoint titers, which was the reciprocal of the greatest dilution that was 0.200 above the background mean of a 1:100 seronegative sample (O.D. = 0.0136). The amino acids that compose the recombinant nucleoprotein fragments of Sin Nombre, Andes and Maporal hantaviruses are highly conserved (Figure 2A), and an antigenicity plot identifies potential binding domains (Figure 2B). To determine the specificity of the ELISA results, we also used the strip immunoblot assay (SIA) that detects rodent antibody against ANDV. The SIA was performed as previously described [15,17,33]. Briefly, 5 µL of rodent blood was used to probe a 1.6 × 5-cm nitrocellulose membrane bearing a horizontal ~0.5-mm × 1.6-mm band containing the affinity-purified recombinant Andes virus N antigen. The membranes were rocked gently overnight at 4 °C at room temperature in a volume of 1 mL of milk-PBST buffer. After washes in detergent buffer, any bound antibodies to N antigen were detected by incubating the membrane with a 1:1000 dilution of goat anti-human IgG alkaline phosphatase-conjugated antibodies and revealed with the 5-bromo, 4-chloro, 3-indolylphosphate/nitroblue tetrazolium (BCIP/ NBT) phosphatase substrate system (KPL Laboratories, MD, USA). The SIA is based on a recombinant protein amplified by PCR using the Andes virus Sout isolated CHI-7913 [36]. For gene expression, *E. coli* BL21 (DE3) cells transformed with pET23a (expression vector) and induced with IPTG (isopropyl-beta-D-thiogalactopyranoside) was used. The coding region of the N protein and the specific primers used for PCR amplification were previously reported [33]. Positive controls are sera of rodents previously captured that resulted seropositive by SIA and PCR positive (using either blood or tissue). Negative controls are also sera of rodents that resulted seronegative by SIA and PCR negative. SIA analyses were performed for all except some samples from the Villarrica locality (total N = 240 samples). In this locality, only the *O. longicaudatus* sera (N = 6; Table 1) were available to perform the SIA. The reactivity of some rodent samples using the SIA that includes seropositives, negative and positive controls is shown in the supplementary material. Bands in the strips are recombinants ANDV-N, ANDV-N modified (new) and SNV-N antigens [8,17], and 1+ and 3+ represent negative rodent sera at dilutions 1:10,000 and 1:1000, respectively.

## Supplementary Files

Supplementary File 1 Supplementary Figure (PDF, 770 KB)
