# Supplementary material for: Rapid Enzyme-Linked Immunosorbent Assay for the Detection of Hantavirus-Specific Antibodies in Divergent Small Mammals
Source: Viruses. 2014 May 6;6(5):2028–37. doi: 10.3390/v6052028 (PMC4036537; doi:10.3390/v6052028)

Supplementary Figure S1. Reactivity of rodent samples using strip immunoassay. Seropositive samples are labeled with "+" (**A**); negative control (NC) and positive control (PC) are shown in (**B**).

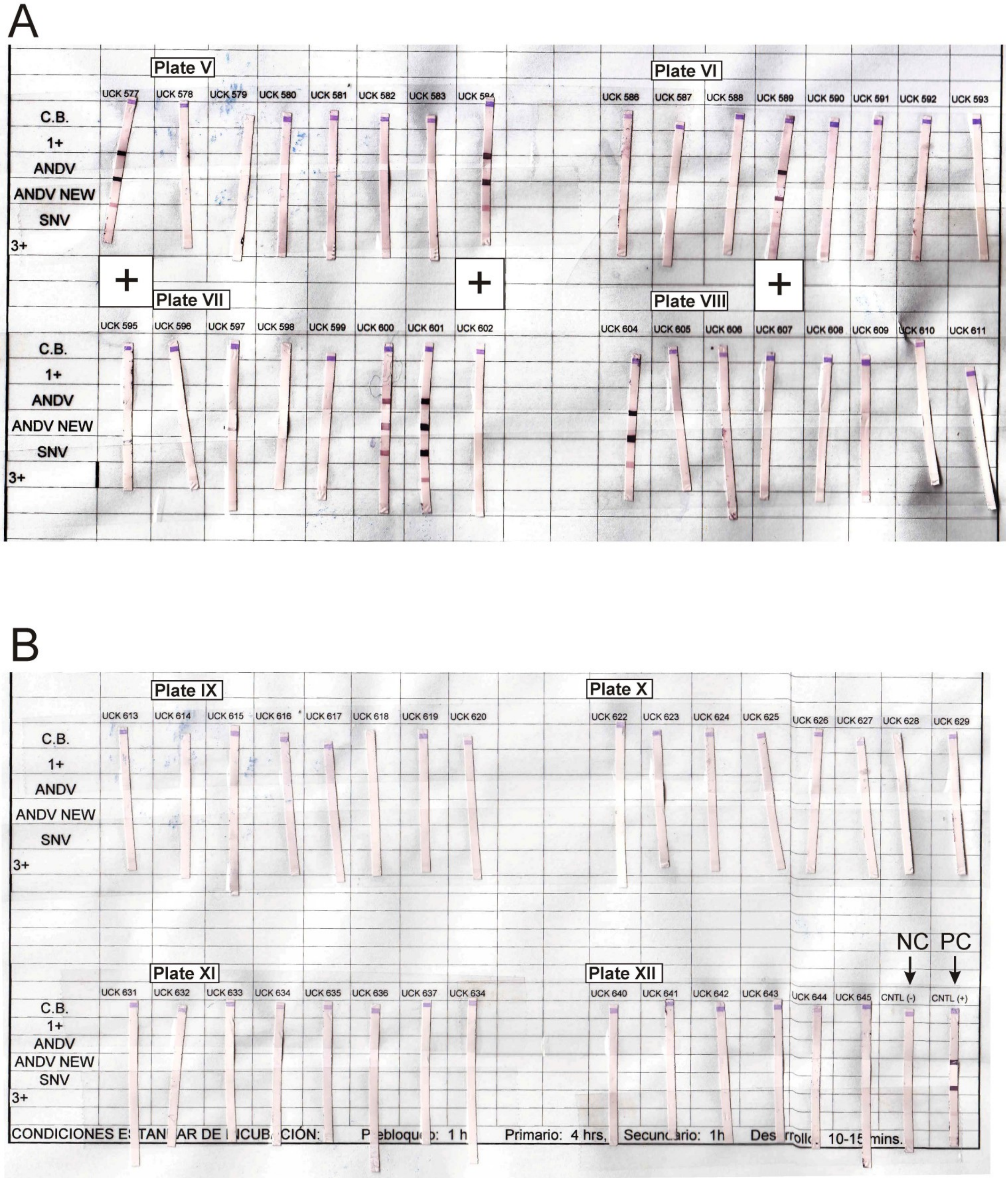

Supplement: Supplementary File 1 — Supplementary Figure (PDF, 770 KB) [file viruses-06-02028-s001.pdf]
